# The promotion of breastfeeding and professional ethics

**DOI:** 10.1186/1824-7288-41-S2-A22

**Published:** 2015-09-30

**Authors:** Riccardo Davanzo

**Affiliations:** 1 Division of Neonatology; Institute for Maternal and Child Health, “IRCCS Burlo Garofolo”, Trieste 34100, Italy

## 

Systematic reviews of the literature have consistently demonstrated that breastfeeding's biological effect extends beyond the nursing period of an infant to when the child is no longer protected by maternal milk. But breastfeeding is also beneficial for the mother, the family and the society [1].

Yet, despite awareness of the positive impact breastfeeding can have on the health of the individual and to a wider extent of a nation, we paediatricians/neonatologists hold a variety of attitudes toward breastfeeding, depending on professional training background (more cure-than care-oriented), professional interest (intensive care rather than physiology and prevention), personal reproductive history (female versus male; own children breastfed rather than bottle-fed).

Whatever the specific interests in medical care or the place breastfeeding holds within a personal value system, the paediatrician/neonatologist will acknowledge its distinct advantages and make an ethical commitment to promoting it [2], within the scope of her/his possibilities, skills, role, competence.

Not every paediatrician is called to advocate breastfeeding; not everyone feels the vocation. What we can do is reflect on our everyday practice: shall we muster the available data in support of breastfeeding? Are we sufficiently informed to avert damaging the reputation and culture of breastfeeding? Do we have conflicts of interest? Are we ready to recognize and to disclosure them?

The same caution that we exercise when faced with routine clinical problems is equally warranted when advising a mother who is trying to decide whether or not to breastfeed her baby. Caution does not mean reticence, censorship of scientific data or prejudice. Instead, such caution is as much a part of professional ethics as are practice based on sound knowledge and the ability to apply and communicate it appropriately in professional contexts.

Eventually, the protection of breastfeeding simply demands and affirms sound professional practice, in other words the practice of quality in health care.
